# Transcriptional and physiological analyses of Fe deficiency response in maize reveal the presence of *Strategy I* components and Fe/P interactions

**DOI:** 10.1186/s12864-016-3478-4

**Published:** 2017-02-13

**Authors:** Laura Zanin, Silvia Venuti, Anita Zamboni, Zeno Varanini, Nicola Tomasi, Roberto Pinton

**Affiliations:** 1 Dipartimento di Scienze Agroalimentari, Ambientali e Animali, University of Udine, via delle Scienze 206, I-33100 Udine, Italy; 2 Dipartimento di Biotecnologie, University of Verona, Ca’ Vignal 1- Strada Le Grazie 15, I-37134 Verona, Italy

**Keywords:** *Zea mays*, Iron, Fe-source, Gene expression, Mineral nutrition, NRAMP, Phosphate transporter, Phosphorous uptake, Root acquisition, Strategy II

## Abstract

**Background:**

Under limited iron (Fe) availability maize, a *Strategy II* plant, improves Fe acquisition through the release of phytosiderophores (PS) into the rhizosphere and the subsequent uptake of Fe-PS complexes into root cells. Occurrence of *Strategy-I*-like components and interactions with phosphorous (P) nutrition has been hypothesized based on molecular and physiological studies in grasses.

**Results:**

In this report transcriptomic analysis (NimbleGen microarray) of Fe deficiency response revealed that maize roots modulated the expression levels of 724 genes (508 up- and 216 down-regulated, respectively). As expected, roots of Fe-deficient maize plants overexpressed genes involved in the synthesis and release of 2’-deoxymugineic acid (the main PS released by maize roots). A strong modulation of genes involved in regulatory aspects, Fe translocation, root morphological modification, primary metabolic pathways and hormonal metabolism was induced by the nutritional stress. Genes encoding transporters for Fe^2+^ (*ZmNRAMP1*) and P (*ZmPHT1;7* and *ZmPHO1*) were also up-regulated under Fe deficiency.

Fe-deficient maize plants accumulated higher amounts of P than the Fe-sufficient ones, both in roots and shoots. The supply of 1 μM ^59^Fe, as soluble (Fe-Citrate and Fe-PS) or sparingly soluble (Ferrihydrite) sources to deficient plants, caused a rapid down-regulation of genes coding for PS and Fe(III)-PS transport, as well as of *ZmNRAMP1* and *ZmPHT1;7*.

Levels of ^32^P absorption essentially followed the rates of ^59^Fe uptake in Fe-deficient plants during Fe resupply, suggesting that P accumulation might be regulated by Fe uptake in maize plants.

**Conclusions:**

The transcriptional response to Fe-deficiency in maize roots confirmed the modulation of known genes involved in the *Strategy II* and revealed the presence of *Strategy I* components usually described in dicots. Moreover, data here presented provide evidence of a close relationship between two essential nutrients for plants, Fe and P, and highlight a key role played by Fe and P transporters to preserve the homeostasis of these two nutrients in maize plants.

**Electronic supplementary material:**

The online version of this article (doi:10.1186/s12864-016-3478-4) contains supplementary material, which is available to authorized users.

## Background

Iron (Fe) deficiency is a yield-limiting factor and a worldwide problem for crop production in many agricultural regions, particularly in aerobic and calcareous soils [1]. Although the total Fe content of soil would be sufficient to meet the needs of plants, most of the Fe in the soil is present in poorly available inorganic forms, especially under aerobic conditions [2]. The level of plant-available Fe in the soil solution is determined by a variety of natural ligands (such as microbial siderophores, humic substances and root exudates) which are able to mobilize Fe from oxides/hydroxides form to Fe(III) chelates [3] and can be used by plants directly or through reduction-based mechanisms. In fact it is well known that plants react to low Fe availability using different adaptive strategies. *Strategy I* is used by all dicots and non‐graminaceous monocots, that respond to Fe‐deficiency by releasing in the rhizosphere protons and chelating/reducing agents (as carboxylates and phenolic compounds) to mobilize Fe from sparingly soluble forms. Afterwards, a ferric-chelate reductase at the surface of root cells (FRO) mediates the reduction of ferric ions to ferrous form [4, 5]. The ferrous ion is, in turn, acquired by roots through a Fe^2+^ transporter belonging to the ZIP metal transporter family (iron regulated metal transporter, IRT) [6].

Graminaceous species, like maize, improve Fe acquisition by releasing phytosiderophores (PS) into the rhizosphere through the PS-efflux transporter TOM1 [7, 8]. Thereafter, the intact Fe(III)-PS complex is taken up through the specific transporter yellow stripe1 (YS1) [9].

In grasses the presence of *Strategy I*-like mechanisms have also been hypothesized [10]. In rice, that is considered to use a combined strategy, the presence of IRT-like protein and natural-resistance-associated-macrophage protein (NRAMP, also known as divalent metal transporter) have been reported to play important roles in the absorption and translocation of ferrous Fe [10–14]. Besides the efflux of phytosiderophores, rice plants possess a dedicated efflux transporter for phenolic compounds (phenolics efflux zero-like transporter, PEZ [15], which is an essential component in order to improve Fe solubilisation from apoplastic space. Genes coding for *ZIP* transporters, putative H^+^-ATPase and Fe^3+^-reductase have been identified in maize [16, 17], although they have not been clearly related to Fe acquisition. Furthermore, OsNRAMP homologous transporter have been identified in maize and reported as responsive element to Fe deficiency [8]. In this way, in monocot species like rice, and putatively in other graminaceous species, the low availability of Fe might be counteracted also by the activation of some molecular components, not exclusively relating to *Strategy II*, involved in the acidification of the rhizosphere and the acquisition of ferrous iron forms. The presence of phenolic compounds and carboxylates in the rhizosphere would help Fe solubilisation, which in turn might be taken up by rice or maize roots via Fe(III)-PS transporter (YS1) or, possibly, as ferrous form by IRT-like/NRAMP transporters.

As it might be expected, a complex response to a specific nutritional stress, including the biosynthesis in plants and the release into the rhizosphere of root exudates, would affect uptake and metabolism of other nutrients. Several studies have shown that a low availability of Fe triggers molecular responses linked to sulphur (S), zinc (Zn) and phosphorous (P) metabolism [18–20]. While the interactions between Fe and S nutrition has been well established [21], there is still poor knowledge about the relationships between Fe and P nutrition in plants, mainly limited to model *Strategy I* plant Arabidopsis [22, 23] and especially in grasses. In rice seedlings, evidence has been provided about antagonistic interactions between Fe and P nutrition. In fact a P deficiency condition resulted in a significant increase in Fe content within plants while the presence of P limited Fe accumulation in plant tissues [20]. In plants, this antagonistic interaction might be due to the mechanisms used by plants to mobilize poorly soluble source of Fe and P from soils, *i.e.* by releasing organic acids and phenolic compounds [24].

Aim of the present work was to study, at physiological and transcriptional level, the Fe deficiency response in maize plants, an economically important crop and a model species for *Strategy II.*

Starting from morphological observations, we characterized the maize root transcriptome. Moreover deep investigations were conducted to evaluate the capability of maize plants to use different Fe sources, such as Fe-Citrate, Fe-PS and the sparingly soluble Ferrihydrite. A relationship between Fe deficiency response and P acquisition processes was also investigated.

## Methods

### Plant material and growth conditions

Maize seeds (*Zea mays* L., inbred line PR33T56, Pioneer Hybrid Italia S.p.A.) were germinated over aerated 0.5 mM CaSO_4_ solution in a dark growth chamber at 25 °C. Five-day-old seedlings were transferred in a continuously aerated Fe-free nutrient solution (containing, μM: NH_4_NO_3_ 1000; CaSO_4_ 500; MgSO_4_ 100; KH_2_PO_4_ 175; KCl 5; H_3_BO_3_ 2.5; MnSO_4_ 0.2; ZnSO_4_ 0.2; CuSO_4_ 0.05; Na_2_MoO_4_ 0.05; buffered solution to pH 6.0 with 2.5 mM 2-(N-morpholino) ethanesulfonic acid (MES)-KOH) and plants were grown for 7 days in a growth chamber under controlled climatic conditions (day/night photoperiod, 16/8 h; radiation, 220 μ Einsteins m^−2^ s^−1^; day/night temperature, 25/20 °C; relative humidity, 70–80%). Thereafter, some 12-day old plants were maintained for a further week in Fe deficiency (Fe-deficient plants, −Fe), while some of 12-day-old maize plants were transferred to a Fe-sufficient nutrient solution containing 100 μM Fe-EDTA (Fe-sufficient plants, +Fe). Nutrient solutions were renewed every three days. During the growth period, light transmittance of leaves was determined on 12-day-old plants, 15-day old plants and 19-day old plants using a portable chlorophyll meter SPAD-502 (Minolta, Osaka, Japan) and presented as SPAD index values.

After 14 days of growing in Fe-free nutrient solution, 19-day-old maize plants showed visual symptoms of Fe deficiency (interveinal yellowing of the young leaves; increase in the diameter of the sub-apical zone and amplified root hair formation). The capability of Fe-deficient and Fe-sufficient plants to acidify the rhizosphere was investigated using the pH indicator bromocresol purple. Thus, whole root systems of intact plants were placed on a 3-mm-thick gel layer containing 0.9% agar, 0.01% bromocresol purple and buffered at pH 5.5 with 1 M KOH.

Elemental analyses were assessed on roots and shoots of 19-day old Fe-deficient and Fe-sufficient plants. Dried samples of roots and leaves were dried, ashed at 550° and digested with H_2_O_2_. Thereafter, samples were diluted with 3.75% HNO_3_ and filtered through a Whatman WCN 0.2 μm membrane filter. Iron, calcium (Ca), potassium (K), magnesium (Mg), manganesium (Mn), sodium (Na), P, S and Zn concentrations (mg Kg^−1^ dry weight) were then determined by ICP-OES (VISTA-MPX, Varian Inc., Palo Alto, USA).

For microarray analyses, roots of Fe-deficient and Fe-sufficient maize plants (19-d old) were harvested three hours after the beginning of light phase. The collected roots were immediately frozen in liquid nitrogen and stored until further processing at −80 °C. The collection was repeated in three independent cultivations and the roots from six plants were pooled for each treatment.

### Microarray analyses

RNA extractions were performed using the Invisorb Spin Plant RNA kit (Stratec Molecular) as reported in the manufacturer’s instructions. Maize roots (70 mg) were homogenized in liquid nitrogen and the powder was mixed with 900 μl of DCT solution and dithiothreitol according to the supplier’s instructions. The RNA quality and quantity were determined using a Bioanalyzer Chip RNA 6000 series II (Agilent).

For the microarray analyses, three independent biological replicates were used, for a total of 6 hybridizations. The cDNA synthesis, labeling, hybridization and washing reactions were performed according to the NimbleGen Arrays User’s Guide (http://www.nimblegen.com/). Each hybridization was carried out on a NimbleGen microarray (Roche, NimbleGen Inc.), representing 59,756 transcripts predicted from the B73 reference genome (http://ftp.maizesequence.org/release-5b/filtered-set/). A complete description of the chip is available at the Gene Expression Omnibus (http://www.ncbi.nlm.nih.gov/geo) under the series entry (GPL17540). The microarray was scanned using an Axon GenePix 4400 (Molecular Devices) at 532 nm (Cy-3 absorption peak) and GenePix Pro7 software (Molecular Devices) according to the manufacturers’ instructions. Images were analyzed using NimbleScan v2.5 software (Roche), which produces Pair Files containing the raw signal intensity data for each probe and Calls Files with normalized expression data- (quantile normalization) derived probe summarization through RMA analysis [25]. Analysis of normalized data (Calls Files) was performed using the open source software of the Bioconductor project (http://www.bioconductor.org/) [26] with the statistical R programming language (http://www.r-project.org/) [27]. Differentially expressed probes were identified by linear model analysis [28] using the LIMMA package and applying Bayesian correction, adjusted *P*-value ≤ 0.05, *n* = 3, FC ≥ |1.5|. All microarray expression data are available at the Gene Expression Omnibus (http://www.ncbi.nlm.nih.gov/geo/) under the series entry (GSE76829).

### Physiological and transcriptional experiments with natural Fe sources: Fe-PS, Fe-Citrate and Ferrihydrite

#### Preparation of Fe-sources

Soluble Fe-sources (Fe-PS and Fe-Citrate) were prepared according to von Wirén et al. [29] by mixing an aliquot of Fe-free (epi-HMA)-containing root exudates collected from Fe-deficient barley plants [30] or citrate (10% excess) with FeCl_3_ [or ^59^FeCl_3_ for radiochemical analyses]. KH_2_^32^PO_4_ solution were prepared to a final concentration 175 μM.

The poorly soluble Fe-sources (Ferrihydrite and Vivianite) were prepared according to previous works [31, 32]. Amorphous Ferrihydrite [or (^59^Fe)Ferrihydrite] was obtained by precipitating Fe(NO_3_)_3_ [or ^59^Fe(NO_3_)_3_] at alkaline pH, with the addition of 1 M KOH [31]. The synthetic (^32^P)Vivianite was obtained through the slow neutralization of 50 mM FeSO_4_ in 35 mM H_3_^32^PO_4_ with 50 mM KOH at room temperature. The pH of the mixture was brought up to 6, where the precipitation of a blue-grey powder occurred. The powder was washed *via* centrifugation with distilled water to remove the presence of salt. One milliliter of suspension containing respectively of Ferrihydrite [or (^59^Fe)Ferrihydrite] (2 μmol Fe) or (^32^P)Vivianite (2 μmol P) were transferred into a dialysis tube (ZelluTrans/Roth 6.0, ∅16 mm, exclusion limit of 8–10 kDa, ROTH, Karlsruhe, Germany).

#### Iron uptake from soluble and poorly soluble sources by maize plants

To evaluate the capability of Fe-deficient plants to use natural Fe sources, physiological and transcriptional experiments were performed on 19-day old intact maize plants.

The day before of the experiment, roots of (18-d old) Fe-deficient plants were washed 3 times with deionized water. Then, two intact plants were transferred to beakers containing 230 ml of a freshly prepared Fe-free nutrient solution. The next day, Fe-PS, Fe-Citrate or Ferrihydrite were added to give a final Fe concentration of 1 μM and the treatment solutions were buffered at pH 6.0 with 10 mM MES-KOH. The photochemical reduction phenomena of Fe in the nutrient solution [33], was limited covering the beakers with black plastic foils during the entire experiment. During the time span of 24 h (after 1, 4, and 24 h) root samples were harvested, frozen in liquid nitrogen and used for the gene expression analyses. The same experimental setup was used for radiochemical analyses where ^59^Fe-sources were supplied to Fe-deficient and Fe-sufficient plants at a final Fe concentration of 1 μM ^59^Fe-PS, ^59^Fe-Citrate or (^59^Fe)Ferrihydrite and at a specific activity of 144 kBq μmol^−1^ Fe (Perkin Elmer, Monza, Italy). At each time-point (1 and 24 h of treatment) plants were transferred into cold deionized water for 10 min in order to stop Fe uptake and remove the excess of ^59^Fe at the root surface. Root apoplastic ^59^Fe pools were removed as described by Bienfait et al. [34], raising roots in a solution (1.2 g l^−1^ sodium dithionite, 1.5 mM 2,2′-bipyridyl and 1 mM Ca(NO_3_)_2_) under N_2_ bubbling gas. After roots and leaves were harvested and collected separately in vials. Root and shoot tissues were oven dried at 80 °C, weighed, ashed at 550 °C, and suspended in 1 M HCl for ^59^Fe determination by liquid scintillation counting. The ^59^Fe accumulation was measured as nanomoles of ^59^Fe and is presented as nmol ^59^Fe g^−1^ root dry weight.

#### Real-time RT–PCR experiments

To validate the microarray results and to analyze in time-course the expression profile of some genes during 24 h of Fe-source treatments, real-time RT–PCR analyses were performed. Total RNA was treated with 1 U μg^−1^ RNA of DNase I (Sigma Aldrich) and cDNA was synthesized from 1 μg of RNA following the application protocol of the manufacturers [42 °C for 1 h with 1 pmol of oligod(T)_23_VN (Sigma Aldrich); 15 U of Prime RNase Inhibitor (Eppendorf); 10 U of M-MulV RNase H– (Finnzymes)]. After RNA digestion with 1 U of RNase A (USB) for 1 h at 37 °C, gene expression analyses were performed by adding 0.16 μl of the cDNA to the real-time RT-PCR complete mix, FluoCycleTM sybr green (20 μl final volume; Euroclone, Pero, Italy), in a C1000 Thermal Cycler-Bio-Rad CFX96 real-time PCR detection system (Bio-Rad). The analyses of real-time RT-PCR result were performed using Bio-Rad CFX Manager v.2 software (Bio-Rad).

Real-time RT-PCRs analyses were performed to evaluate the expression level of most interesting identified by microarray and the modulation of these genes was monitored during the time span of Fe-treatments. The primers were designed using Primer3 software [35, 36] and they were synthesized by Sigma Aldrich (gene model ID, forward and reverse primer sequences 5’- -3’): *ZmYS1* (GRMZM2G156599, AGGAGACAAGAACGCAAGGA and ACTGAACAAAGCCGCAAACT), *ZmTOM1* (GRMZM2G063306, AGGAGTTCTTCTTCGTCGCA and GCACCAAGAAAACCAGCGTA), *ZmOPT7* (GRMZM2G421491, TCGTCTGGAAGGAGGAGATG and CGGTTGCTGGTTAGTGGTG), *ZmNRAMP1* (GRMZM2G178190, GGAGAATTATGGCGTGAGGA and ACCACCAAACCGATCAGAAG), *ZmFerritin* (GRMZM2G325575, GATGCTGCTTGAGGAAGAGG and CCGACCCAGAGTTGTCAGTT), *ZmPHT1;7* (GRMZM2G112377, TCCTGATGATGACGGTGTTC and GAAGTTGGCGAAGAAGAAGG).

The analyses of real-time RT-PCR results were performed using Bio-Rad CFX Manager v.2 software (Bio-Rad) and R (version 2.9.0; http://www.r-project.org/) with the qPCR package (version 1.1-8; http://www.dr-spiess.de/qpcR.html). Efficiencies of amplification were calculated following the authors’ indications [37]. Real-time RT–PCR results were validated using two housekeeping genes, *ZmGAPDH* (GRMZM2G046804, CCTGCTTCTCATGGATGGTT and TGGTAGCAGGAAGGGAAGCA) and *ZmTUA* (GRMZM2G152466, AGGTCATCTCATCCCTGACG and TGAAGTGGATCCTCGGGTAG). Data were normalized with respect to the transcript level of the housekeeping genes using the 2^–ΔΔCT^ method, where ΔΔC_T_ = (C_T,Target_ – C_T,HK_)_Time x_ – (C_T,Target_ – C_T,HK_) _Time 0_ [38].

#### Phosphorous (^32^P) uptake from soluble and poorly soluble sources by maize plants

To investigate the effect of natural Fe-sources on the acquisition of P in plants, (19-d old) Fe-deficient and Fe-sufficient maize plants were treated with three unlabeled-natural Fe sources (Fe-Citrate, Fe-PS and Ferrihydrite), as reported above. Moreover, nutrient solution contained also (^32^P)-radiolabeled sources, as the soluble source KH_2_^32^PO_4_ or the poorly soluble source (^32^P)Vivianite.

Before starting the assay, the roots were washed 2 times for 5 min in 0.5 mM CaSO_4_, in order to remove the apoplastic component. Afterwards the plants were transferred into the uptake solution, buffered to pH 6.0 with 10 mM MES-KOH and containing phosphorus supplied as KH_2_^32^PO_4_ or (^32^P)Vivianite labeled with ^32^P in an amount equal to 2.47 kBq μmol^−1^. After 24 h, root and leaves of maize plants were sampled and the P accumulation in the tissues was determined. Root samples were transferred for 5 min in a cold washing solution. In this way, the apoplastic radioactive component was removed and the uptake of ^32^P was blocked. Roots and leaves were harvested and collected separately and oven dried at 80 °C, weighed, ashed at 550 °C, and suspended in 1 M HCl. The determination of ^32^P concentration was performed by liquid scintillation counting. The ^32^P accumulation, measured as nanomoles of ^32^P and is presented as nmol ^32^P g^−1^ root dry weight.

### Statistical analyses

Physiological and transcriptomic analyses were performed on three independent biological replicates obtained from independent experiments (*n* = 3); for each sample a pool of six plants was used. Statistical significance was determined by one-way analysis of variance (ANOVA) using Student–Newman–Keuls test (*P* <0.05, *n* = 3). Statistical analyses were performed using SigmaPlot Version 12.0 software. Statistical analysis of microarray data was performed using linear model analysis [28] of the LIMMA package after Bayesian correction with Bioconductor software, adjusted *P*-value ≤ 0.05, *n* = 3, FC ≥ |1.5| (for details see the ‘Microarray analyses’ section).

## Results

### Morphological comparison between Fe-deficient and Fe-sufficient maize plants

**Fig. 1:**
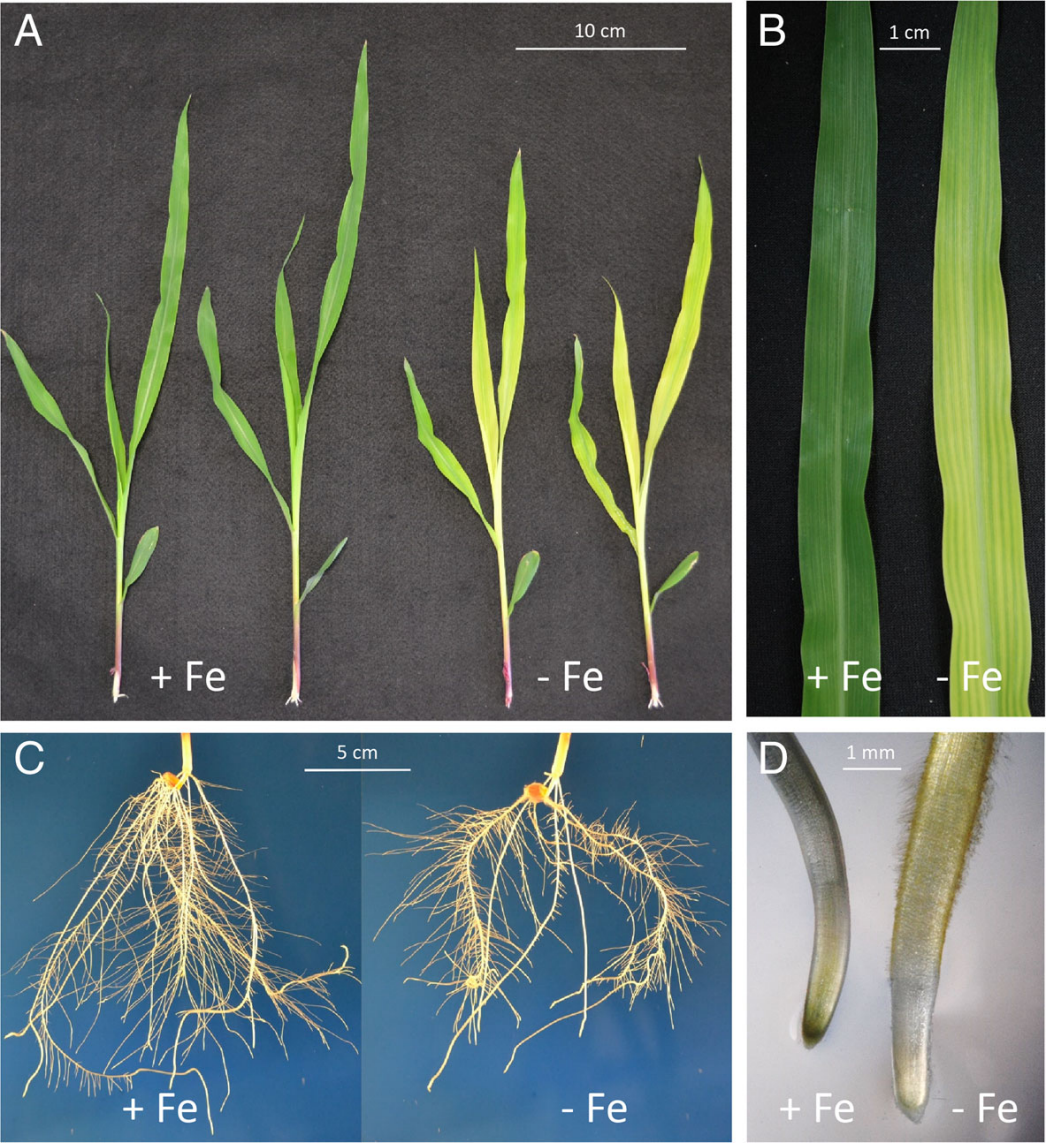
Shoot and root apparatus of maize plants grown under different Fe-supply conditions. **a**, shoots of Fe-sufficient plants (left) and shoots of Fe-deficient plants (right); **b**, leaf details of Fe-sufficient (left) and Fe-deficient (right) plants. **c**, roots of Fe-sufficient plants (left) and roots of Fe-deficient plants (right); **d**, details of root tips of Fe-sufficient (left) and Fe-deficient (right) plants after soaking roots with pH indicator (bromocresol purple), as indicated in Methods

**Fig. 2:**
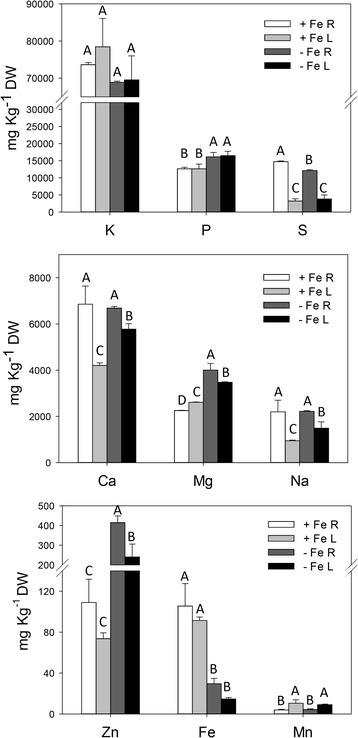
Macro- and micronutrient concentration [mg Kg^−1^ dry weight (DW)] in roots (R) and leaves (L) of 19**-**day-old maize plants grown under Fe sufficiency (+Fe) or Fe deficiency (−Fe). Data are means + SD of three independent experiments (capital letters refer to statistically significant differences among samples for a single nutrient, ANOVA Holm–Sidak, *P <* 0.05, *n* = 3)

At the end of the growing period, Fe-deficient plants showed typical symptoms of Fe-starvation (Fig. 1, Additional file 1: Figure S1). Indeed Fe-deficient plants showed visible interveinal yellowing of the young leaves (Fig. 1a,b) with a reduction in the chlorophyll content (SPAD index value, Additional file 1: Figure S1). The nutritional stress induced changes also in the root architecture: −Fe plants showed shorter roots and an overall reduction in the morphometric parameters of the root apparatus (Additional file 2: Table S1). Furthermore, −Fe roots showed an increased in the diameter of the sub-apical root zone (Fig. 1c,d) with high density of root hairs and a concomitant increased acidification of the external solution (Additional file 1: Figure S2). The multi-element analysis showed lower amounts of Fe in Fe-deficient plants as compare to sufficient ones, and, only in roots, low amounts of S. On the other hand, deficient plants accumulated higher amounts of Mg, Zn, P and, only in leaves, Ca and Na (Fig. 2).

### Root transcriptomic response to Fe deficiency

In order to investigate the Fe-deficient response in maize, microarray analyses were performed on samples of Fe-deficient and Fe-sufficient roots. Analyses were performed using the maize chip 12_135K Arrays from Roche NimbleGen (http://www.nimblegen.com) which allowed the monitoring of 59,756 transcripts. Transcriptional profiles were identified and statistically analyzed by Linear Models for MicroArray (LIMMA) [28], adjusted *P*-value ≤ 0.05, *n* = 3, fold change (FC) ≥ |1.5|. Results indicate that Fe starvation induced changes in the root transcriptome involving 724 transcripts, 508 of which were up-regulated while 216 were down-regulated (Additional file 3: Table S2).

The differentially expressed transcripts were annotated based on the description file provided by Phytozome (*Zea mays* 284_6a JGI download) and clustered under functional categories according to the biological process of Gene Ontology (GO, http://www.geneontology.org). Referring to the total number of modulated transcripts, the most abundant categories were “metabolic process” (36% of modulated transcripts), “biological regulation” (11%), “localization” (10%) and “cellular process” (6%), while 33% of modulated transcripts showed unknown function. The main GO categories included more up-regulated than down-regulated transcripts (Additional file 1: Figure S3).

**Fig. 3:**
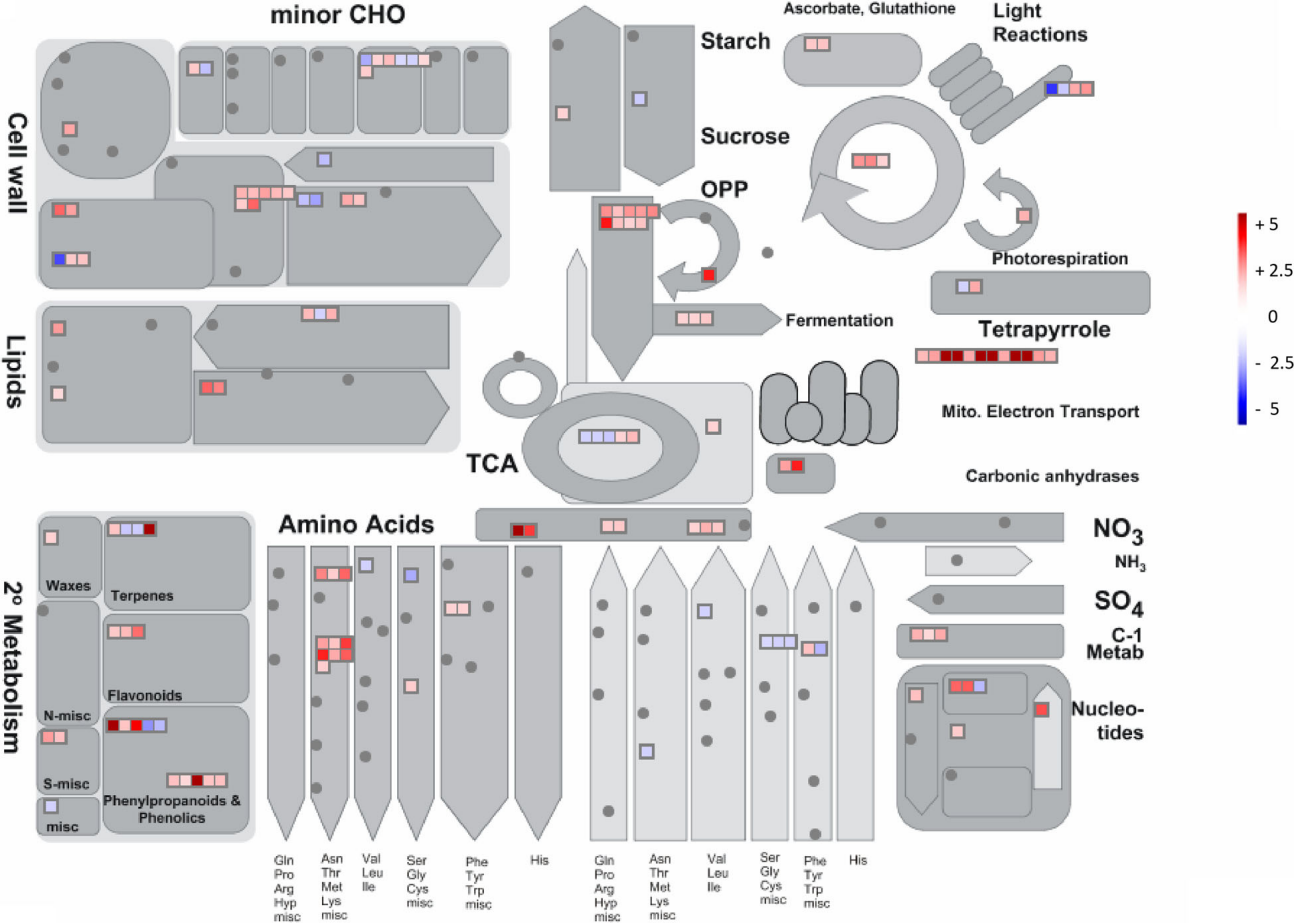
Transcriptional modulation of genes involved in the cell metabolism by Fe deficiency. Color scale refers to the fold change values of differentially expressed transcripts: *red* color refers to those transcripts positively regulated by Fe deficiency, while in *blue* are transcripts negatively regulated by Fe deficiency (FC ≥ |1.5|, adjusted *P*-value ≤ 0.05, *n* = 3)

Global functional analysis based on ‘MapMan bins’ indicated that Fe deficiency modulated transcripts involved in glycolysis, mitochondrial electron transport/ATP synthesis, hormone metabolism, amino acid metabolism, abiotic stress, secondary metabolism, signaling and membrane transport functions (Fig. 3, Additional file 1: Figure S4).

Among transcripts involved in glycolysis, fermentation and TCA cycle, Fe starvation up-regulated those coding for a glyceraldehyde-3-phosphate dehydrogenase (GADPH), phosphofructokinase (PFK), fructose-bisphosphate aldolase (FBP aldolase) and phosphoenolpyruvate carboxylase (PEPC), pyruvate decarboxylase (PDC), aldehyde dehydrogenase (ADH), lactate/malate dehydrogenase (LDH), citrate synthase (CS), isocitrate dehydrogenase (IDH), NADP-malic enzyme (NADP-ME, Fig. 3, Additional file 3: Table S2).

In agreement with morphological observation at the root level, several transcripts involved in the cell wall formation and lipid synthesis were modulated by Fe availability (Fig. 3).

**Fig. 4:**
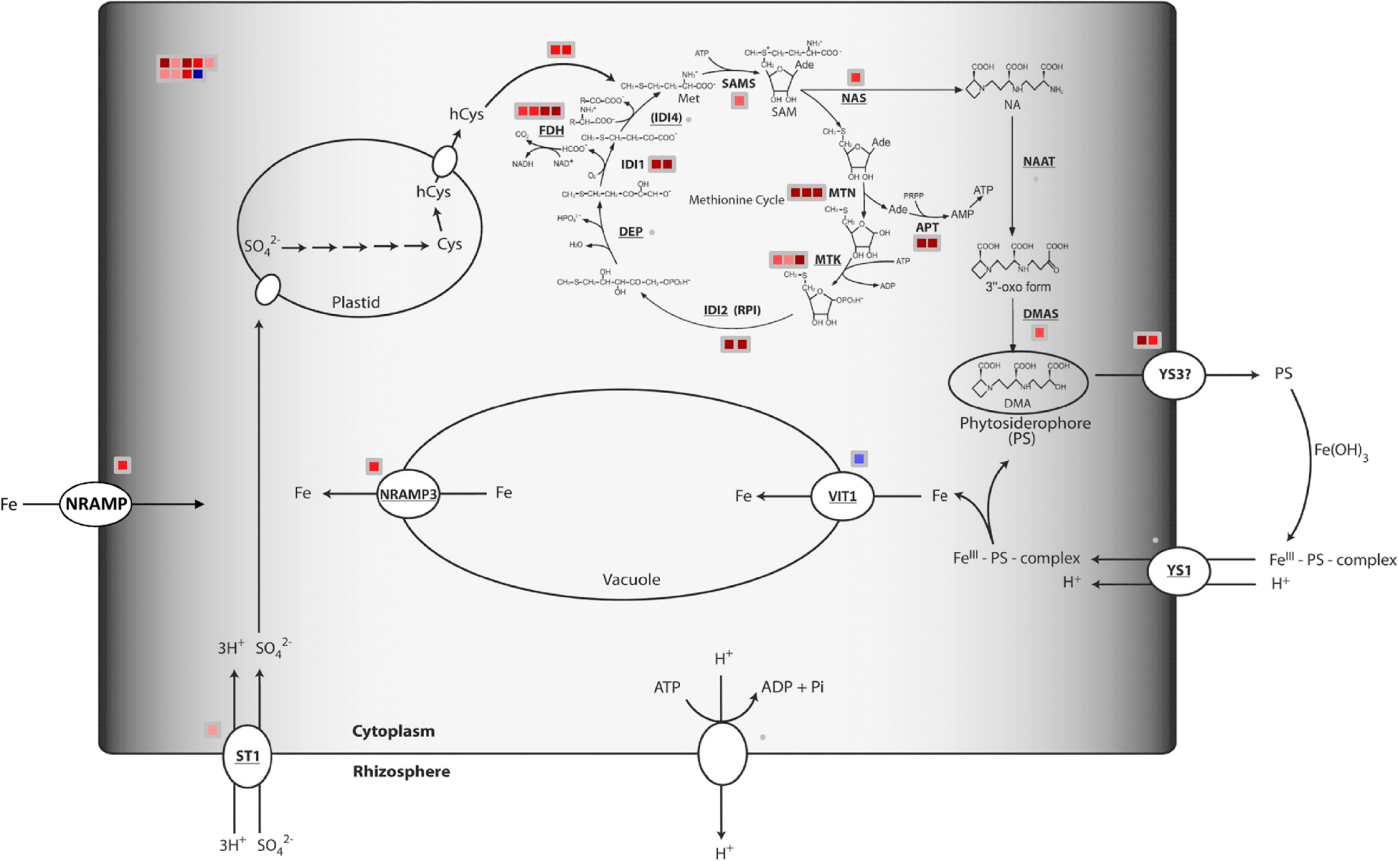
Transcriptional changes in roots of genes involved in the Methionine cycle/DMA synthesis and Fe acquisition by Fe deficiency. Abbreviations: YS1 = yellow stripe 1; YS3 = yellow stripe 3 (*ZmTOM1* gene*,* GRMZM2G063306); ST1 = Sulfate transporter 1; SO_4_^2−^ = sulfate; Cys = cysteine; hCys = homo-cysteine; FDH = formate dehydrogenase; DEP = methylthioribulose-1-phosphate dehydratase-enolase-phosphatase; IDI1 = 2-keto-methylthiobutyric-acid forming enzyme; IDI4 = putative aminotransferase catalyzing the synthesis of methionine from 2-keto-methylthiobutyric acid; SAMS = S-adenosyl-methionine synthase; MTN = methylthioadenosine/S-adenosyl homocysteine nucleosidase; MTK = methylthioribose kinase; IDI2 = eukaryotic initiation factor 2B-like methylthioribose-1-phosphate isomerase; *NAS* = nicotinamine synthase; NAAT = nicotianamine amino-transferase; DMAS = 2’-deoxymugineicacid synthase; APT = Adenosin phosphoribosyltransferase; VIT1 = vacuolar iron transporter 1; NRAMP = natural resistance associated macrophage protein; Regulation = GRMZM2G057413 (homology to *OsIRO2),* GRMZM2G350312 (homology to *OsIRO3*)*,* GRMZM2G107672 (homology to AtbHLH29), GRMZM2G133675 (homology to *AtPYE*). Color scale refers to the fold change values of differentially expressed transcripts: *red* color refers to those transcripts positively regulated by Fe deficiency, while in *blue* are transcripts negatively regulated Fe deficiency. Adapted from Urbany et al. [39] and Benke et al. [64]

In Fe-deficient roots also some pathways for the synthesis of amino acids were induced, especially those for the synthesis of methionine and its derivatives (as phytosyderophores), which play a crucial role in the *Strategy II*. To provide a clearer representation, the modulated transcripts were mapped on a custom pathway [39] for phytosiderophore synthesis and iron transport in *Strategy II* plants (Fig. 4). Iron starvation induced the expression of many transcripts involved in the pathway for the synthesis and release in the soil of deoxymugineic acid (DMA, Fig. 4).

**Fig. 5:**
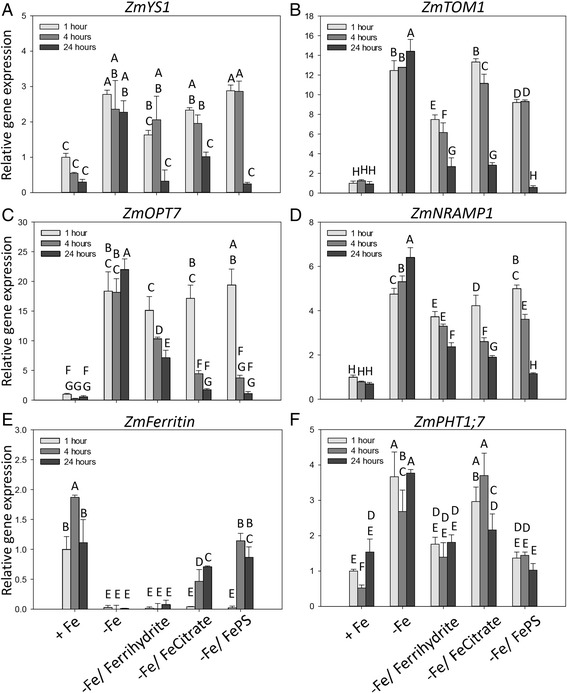
Real-time RT-PCR analyses of gene transcript levels in maize roots. Nineteen-day-old maize plants were grown under Fe sufficiency (+Fe) or Fe deficiency (−Fe); these latter were then supplied with three different Fe-sources: Ferrihydrite, Fe-Citrate or Fe-PS (−Fe/Ferrihydrite, −Fe/Fe-Citrate or –Fe/Fe-PS, respectively). Root samples were harvested after 1, 4 and 24 h from the beginning of the experiment. Analyzed genes: **a**, *ZmYS1* (GRMZM2G156599); **b**, *ZmTOM1* (GRMZM2G063306); **c**, *ZmOPT7* (GRMZM2G421491); **d**, *ZmNRAMP1* (GRMZM2G178190); **e**, *ZmFerritin* (GRMZM2G325575); **f**, *ZmPHT1;7* (GRMZM2G112377). Real-time RT–PCR results were validated using two housekeeping genes, *ZmGAPDH* and *ZmTUA,* in this figure results normalized on *ZmGAPDH* are shown. Gene mRNA levels were normalized with respect to the mean transcript level of the housekeeping gene *ZmGAPDH*; relative changes in gene transcript levels were calculated on the basis of the mean transcript level of *ZmGAPDH* in Fe-sufficient roots at 1 h (relative gene expression = 1). Data are means of three independent biological replicates + SD (ANOVA Holm–Sidak, *P* < 0.05, *n* = 3)

Several transcripts encoding transporters were modulated in response to Fe status. In particular our data identified some transcripts putatively involved in the acquisition/translocation of iron (encoding TOM1, VIT1, NRAMP1, OPT7 and YS-like transporters), sulphate/molybdate (MOT1), zinc (ZIFL1, ZIFL2, ZIP4) and even phosphate (PHO1, PHT1;7, Table 1). The modulation of five differentially expressed transcripts in response to Fe deficiency was confirmed through real-time RT-PCR experiments (Fig. 5).

**Table 1:** List of modulated transcripts involved in the Fe acquisition processes and reported in the Results and Discussion sections by the comparison of transcriptomic profiles of Fe-deficient roots with profile of Fe-sufficient ones, −*Fe* vs *+ Fe* comparison (Fold Change (FC) ≥ |1.5|, adjusted *P*-value ≤ 0.05, *n* = 3)

#^a^	Transcript ID^b^	FC^c^	Description^d^	Protein symbol^e^	Rice gene ID^f^
*Strategy II genes*					
1	GRMZM2G050108_T01	2.21	nicotianamine synthase 4	NAS	LOC_Os07g48980.1
2	GRMZM2G060952_T01	2.05	NAD(P)-linked oxidoreductase superfamily protein	DMAS	LOC_Os03g13390.2
3	GRMZM2G063306_T01	3.83	zinc induced facilitator-like 1	TOM1	LOC_Os11g04020.1
4	GRMZM2G063306_T02	2.31	zinc induced facilitator-like 2	TOM1	LOC_Os11g04020.1
*Methionine cycle and DMA synthesis*					
5	GRMZM2G049811_T01	2.20	formate dehydrogenase	FDH	LOC_Os06g29180.1
6	GRMZM2G054123_T01	1.94	S-adenosylmethionine synthetase family protein	SAMS3	LOC_Os01g22010.1
7	GRMZM2G113873_T01	1.77	cystathionine gamma-synthase, putative, expressed	CYS1	LOC_Os03g25940.4
8	GRMZM2G131907_T01	3.24	adenine phosphoribosyl transferase 1	APT1	LOC_Os12g39860.1
9	GRMZM2G131907_T02	3.12	adenine phosphoribosyl transferase 1	APT1	LOC_Os12g39860.1
10	GRMZM2G152470_T01	2.35	homocysteine methyltransferase 2	HMT2	LOC_Os12g41390.1
11	GRMZM2G152470_T03	2.43	homocysteine methyltransferase 2	HMT2	LOC_Os12g41390.1
12	GRMZM2G165998_T01	2.90	RmlC-like cupins superfamily protein	ARD2	LOC_Os03g06620.1
13	GRMZM2G165998_T02	3.03	acireductone dioxygenase 1	ARD1	LOC_Os03g06620.1
14	GRMZM2G171111_T01	3.20	Phosphorylase superfamily protein	MTN2	LOC_Os06g02220.1
15	GRMZM2G171111_T02	3.53	methylthioadenosine nucleosidase 1	MTN1	LOC_Os06g02220.1
16	GRMZM2G171111_T04	3.77	methylthioadenosine nucleosidase 1	MTN1	LOC_Os06g02220.1
17	GRMZM2G362021_T01	2.29	formate dehydrogenase	FDH	LOC_Os06g29180.1
18	GRMZM2G464137_T01	2.03	S-methyl-5-thioribose kinase	MTK	LOC_Os04g57400.1
19	GRMZM2G464137_T02	1.73	S-methyl-5-thioribose kinase	MTK	LOC_Os04g57400.1
20	GRMZM2G464137_T03	3.11	S-methyl-5-thioribose kinase	MTK	LOC_Os04g57400.1
21	GRMZM5G891282_T01	3.82	ribose-5-phosphate isomerase 2	RPI2	LOC_Os04g24140.1
22	GRMZM2G057506_T02	1.67			LOC_Os01g72360.1
23	GRMZM2G131907_T02	3.12	adenine phosphoribosyl transferase 1	APT1	LOC_Os12g39860.1
24	GRMZM2G131907_T01	3.24	adenine phosphoribosyl transferase 1	APT1	LOC_Os12g39860.1
25	GRMZM2G418005_T02	1.57	formate dehydrogenase	FDH	LOC_Os06g29180.1
*Transcription factors*					
26	GRMZM2G057413_T01	3.20	basic helix-loop-helix DNA-binding domain containing protein, expressed	IRO2	LOC_Os01g72370.1
27	GRMZM2G057413_T02	10.07	basic helix-loop-helix DNA-binding domain containing protein, expressed	IRO2	LOC_Os01g72370.1
28	AC193786.3_FGT005	2.44	basic helix-loop-helix DNA-binding domain containing protein, expressed	IRO4	LOC_Os01g72370.1
29	GRMZM2G350312_T01	1.63	basic helix-loop-helix DNA-binding domain containing protein, expressed	IRO3	LOC_Os03g26210.1
30	GRMZM2G350312_T03	1.68	basic helix-loop-helix DNA-binding domain containing protein, expressed	IRO4	LOC_Os03g26210.1
31	GRMZM2G350312_T04	1.62	basic helix-loop-helix DNA-binding domain containing protein, expressed	IRO5	LOC_Os03g26210.1
32	GRMZM2G107672_T01	2.58	FER-like regulator of iron uptake	FER-like	LOC_Os04g31290.1
33	GRMZM5G898290_T01	1.62	NAC domain containing protein 80	NAC	LOC_Os02g36880.4
34	GRMZM5G898290_T02	2.32	NAC domain containing protein 80	NAC	LOC_Os02g36880.4
*Other genes*					
35	GRMZM2G029135_T01	1.62	1-aminocyclopropane-1-carboxylate synthase	ACCS	LOC_Os01g08270.1
36	AC148152.3_FGT005	2.25	1-aminocyclopropane-1-carboxylate oxidase	AACO	LOC_Os03g48430.1
37	GRMZM2G041418_T01	50.74	alternative NAD(P)H dehydrogenase	ADH2	LOC_Os07g37730.1
*Transporters*					
38	GRMZM2G178190_T01	2.31	NRAMP metal ion transporter	NRAMP1	LOC_Os03g11010.1
39	GRMZM2G112377_T01	2.30	phosphate transporter 1;7	PHT1;7	LOC_Os08g45000.1
40	GRMZM5G891944_T01	2.19	phosphate transporter 1, putative, expressed	PHO1;H1	LOC_Os01g02000.1
41	GRMZM2G064657_T01	2.63	phosphate transporter 1, putative, expressed	PHO1;H1	LOC_Os06g29790.1
42	GRMZM2G421491_T01	11.76	oligopeptide transporter, putative, expressed	OPT7	LOC_Os03g54000.1

^a^Number of transcript

^b^Gene identifiers as retrieved from http://www.maizesequence.org - reference annotation file ZmB73_5b

^c^Fold Change (FC) of -Fe *vs* + Fe (adjusted *P*-value ≤ 0.05, *n* = 3)

^d^Description of putative function (database information from http://www.maizesequence.org)

^e^Symbol of maize protein

^f^Accession number for the homologous sequences from *Oryza sativa* (database information from http://www.maizegdb.org)

### Expression pattern of transcripts encoding transporters in response to Fe supply

The response of maize plants to Fe deficiency was further evaluated analyzing the capability of Fe-deficient plants to use three different natural Fe-sources (Fe-PS, Fe-Citrate or Ferrihydrite) provided at a concentration (1 μM Fe) conceivably present in soils affected by low Fe availability.

By real-time RT-PCR, the expression of some transcripts relating to Fe acquisition were analyzed in Fe-deficient roots exposed for up to 24 h to the different treatments (Fig. 5).

The genes coding for the PS efflux transporter and for Fe(III)-PS influx transporter, *ZmTOM1* and *ZmYS1* respectively, were positively modulated by Fe deficiency. On the other hand, when Fe-sources were supplied to Fe-deficient plants, the expression levels of these transcripts decreased considerably reaching, after 24 h, mRNA amounts comparable to those of Fe-sufficient plants (Fig. 5a,b). A similar behavior was also observed for *ZmOPT7* and *ZmNRAMP1*, two genes putatively involved in the response to Fe deficiency. The expression of these two genes was already modulated after 4 h of treatment with the Fe-sources (Fig. 5c,d).

Moreover differences in the expression levels could be appreciated comparing the treatments. After 24 h of Fe supply, maize roots down-regulated most transcripts reaching expression levels comparable to those recorded under Fe sufficiency, this effect being particularly evident when Fe-PS was supplied to the nutrient solution (Fig. 5a,b,c,d,f). Concerning *ZmOPT7*, Fe-deficient maize roots were more responsible when soluble Fe sources (Fe-PS and Fe-Citrate) were supplied to nutrient solution. After 24 h of treatment, deficient plants treated with Fe-PS or Fe-Citrate showed transcript levels comparable to those recorded in Fe-sufficient plants, while higher values were recorded for *ZmOPT7* and *ZmNRAMP1* when the poorly soluble source Ferrihydrite was supplied (Fig. 5c).

As observed by microarray analyses, Fe deficiency induced also the expression of some transcripts involved in phosphate transport. In particular the expression of *ZmPHT1;*7, a transcript coding for a phosphate transporter was analyzed when maize plants were treated with the three Fe-sources. Confirming transcriptomic data, the Fe-starved roots accumulated higher amounts of *ZmPHT1;7* transcript than Fe-sufficient roots. Supply of Fe-PS or Ferrihydrite rapidly decreased the expression of *ZmPHT1;7*, while with Fe-Citrate this effect was evident only after 4 h of treatment (Fig. 5f).

Finally, we evaluated the expression of a gene *(ZmFerritin)* encoding a ferritin protein, that was highly expressed under Fe sufficiency. The addition of 1 μM of soluble Fe (as Fe-Citrate or Fe-PS) to the Fe-free nutrient solution induced a gradual increase in the gene expression. On the other hand, a low expression of the gene was recorded in roots of Fe-deficient and Ferrihydrite-treated plants (Fig. 5e).

### Iron (^59^Fe) uptake from different Fe sources

In order to highlight the ability of maize plants to use the natural Fe-sources, Fe uptake experiments were performed supplying ^59^Fe-PS, ^59^Fe-Citrate or (^59^Fe)Ferrihydrite for 24 h.

The accumulation of ^59^Fe by the whole plants is reported in Table 2. After 1 h, Fe-deficient plants treated with ^59^Fe-PS accumulated higher levels of ^59^Fe than the Fe-sufficient ones. A lower ^59^Fe amount was accumulated when plants were supplied with the other two ^59^Fe-sources (^59^Fe-Citrate or (^59^Fe)Ferrihydrite) and no significant difference was recorded comparing the two growth conditions (+Fe and -Fe).

**Table 2:** Iron-(^59^Fe) accumulated in maize plants and percentage distribution in leaves and roots

	^59^Fe in plants (nmol g^−1^ DW roots)		^59^Fe in roots (%)		^59^Fe in leaves (%)	
	+Fe	-Fe	+Fe	-Fe	+Fe	-Fe
*1 h*						
(^59^Fe)Ferrihydrite	4.94 ± 1.34 e	7.36 ± 1.58 D	97.1	98.5	2.9	1.5
^59^Fe-Citrate	8.31 ± 1.20 e	14.40 ± 4.15 D	79.1	96.2	20.9	3.8
^59^Fe-PS	120.51 ± 25.67 c	177.24 ± 7.70 B*	95.0	99.8	5.0	0.2
*24 h*						
(^59^Fe)Ferrihydrite	43.24 ± 6.14 d	59.33 ± 5.10 C*	85.6	96.2	14.4	3.8
^59^Fe-Citrate	323.46 ± 111.45 b	694.37 ± 39.57 A*	28.3	69.2	71.7	30.8
^59^Fe-PS	870.15 ± 31.93 a	587.50 ± 63.37 A*	27.1	45.3	72.9	54.7

The plant ability to accumulate Fe was evaluated by ^59^Fe uptake experiments on Fe-sufficient (+Fe) and Fe-deficient (−Fe) plants treated for 1 and 24 h with three labelled ^59^Fe-sources: (^59^Fe)Ferrihydrite, ^59^Fe-Citrate or ^59^Fe-PS. Data are means ± SD of three independent experiments. *Small letters,* refer to statistically significant differences among Fe-sufficient plants; *capital letters,* refer to statistically significant differences among Fe-deficient plants, *asterisks*, refer to statistically significant differences between the two growth condition (−Fe *vs* + Fe). ANOVA Holm–Sidak, *P* < 0.05, *n* = 3). DW, dry weight

Most of the accumulated ^59^Fe was retained in the roots, with the exception of Fe-sufficient plants treated with ^59^Fe-Citrate (Table 2, Additional file 1: Figure S5).

At the end of the experiment (24 h), the amount of ^59^Fe in whole plants was dependent on the Fe-source supplied to nutrient solution and on the nutritional status. In Fe-sufficient plants ^59^Fe accumulation increased following the sequence: (^59^Fe)Ferrihydrite < ^59^Fe-Citrate < ^59^Fe-PS. Fe-deficient plants ^59^Fe accumulated almost the same amount of ^59^Fe from the soluble sources; a much lower amount of ^59^Fe was accumulated in plants treated with Ferrihydrite ((^59^Fe)Ferrihydrite < ^59^Fe-Citrate = ^59^Fe-PS, Table 2).

In Fe-sufficient plants translocation accounted for *ca*. 70% of the absorbed ^59^Fe when ^59^Fe-Citrate and ^59^Fe-PS were supplied, while only *ca*. 15% in presence of (^59^Fe)Ferrihydrite.

Iron-deficient plants showed a translocation rate of about 55% and 31% when supplied with ^59^Fe-PS or ^59^Fe-Citrate, respectively, while only about 4% when the poorly soluble source ((^59^Fe)Ferrihydrite) was provided (Table 2).

### Phosphorous (^32^P) uptake as affected by Fe nutrition

To investigate a possible influence of Fe-nutritional status of plants on phosphate uptake, the ^32^P accumulation was measured after a 24-h exposure to the three different Fe-sources (Ferrihydrite, Fe-Citrate or Fe-PS) in presence of two different ^32^P sources: KH_2_^32^PO_4_, as a soluble form, and (^32^P)Vivianite, as a poorly soluble source (Table 3). Fe-sufficient plants supplied with KH_2_^32^PO_4_ showed a higher accumulation of ^32^P and a higher translocation rates as compared to the Fe-deficient ones. In the sufficient plants ^32^P accumulation was unaffected by the Fe treatment.

**Table 3:** Phosphorous-(^32^P) accumulated in maize plants and percentage distribution between leaves and roots

	^32^P in plants (μmol g^−1^ DW roots)		^32^P in roots (%)		^32^P in leaves (%)	
	+Fe	-Fe	+Fe	-Fe	+Fe	-Fe
*KH*_*2*_^*32*^ *PO*_*4*_						
Ferrihydrite	90.93 ± 5.46 a	37.74 ± 5.66 B*	49.2	69.5	50.8	30.5
Fe-Citrate	104.00 ± 16.36 a	63.51 ± 3.43 A*	37.6	62.6	62.4	37.4
Fe-PS	97.36 ± 8.66 a	56.17 ± 2.09 A*	44.7	68.1	55.3	31.9
*(* ^*32*^ *P)Vivianite*						
Ferrihydrite	0.11 ± 0.03 b	0.13 ± 0.01 C	76.0	88.7	24.0	11.3
Fe-Citrate	0.11 ± 0.01 b	0.13 ± 0.01 C	76.9	88.3	23.1	11.7
Fe-PS	0.11 ± 0.01 b	0.19 ± 0.02 C*	76.3	91.4	23.7	8.6

The plant ability to accumulate *P* was evaluated by ^32^P uptake experiments on Fe-sufficient (+Fe) and Fe-deficient (−Fe) plants. Up to 24 h, −Fe and + Fe plants were treated with three unlabelled Fe-sources (Ferrihydrite, Fe-Citrate or Fe-PS) provided in conjunction with two different labelled ^32^P-sources: KH_2_^32^PO_4_ or (^32^P)Vivianite. Data are means ± SD of three independent experiments. *Small letters,* refer to statistically significant differences among Fe-sufficient plants; *capital letters,* refer to statistically significant differences among Fe-deficient plants, *asterisks*, refer to statistically significant differences between the two growth condition (−Fe *vs* + Fe). ANOVA Holm–Sidak, *P* < 0.05, *n* = 3). DW, dry weight

Fe-deficient plants showed similar levels of ^32^P accumulation from KH_2_^32^PO_4_ when supplied with Fe-Citrate or Fe-PS, but almost double as compared to Ferrihydrite supply.

Uptake from (^32^P)Vivianite was extremely low and almost unaffected by the nutritional status and Fe-source supplied (Table 3, Additional file 1: Figure S6).

## Discussion

### Characterization of Fe deficiency responses in maize roots

Morphological and transcriptome response to Fe deficiency was analyzed in 19-day-old maize plants. Besides typical symptoms of Fe deficiency in leaves (interveinal chlorosis), also roots showed morphological changes, such as a thicker subapical root zone with proliferation of root hairs; these changes, together with an intense acidification of the root external medium, have been widely reported for dicots [40, 41]. However, proliferation of lateral roots has also been reported in maize [42].

Microarray analysis of more than 60,000 maize transcripts revealed that Fe deficiency modulated in roots about 700 transcripts, 508 of which were up-regulated while 216 were down-regulated. This result is in agreement with previous observations showing that the plant response to Fe deficiency is based on the modulation of a narrow set of transcripts [39, 41, 43, 44].

Considering the crucial role of Fe as cofactor of a wide range of enzymes, many genes encoding for cytochromes, catalase, peroxidase isozymes, ferredoxin, and isozymes of superoxide dismutase were found down-regulated by Fe deficiency in maize roots.

On the other hand, grasses counteract Fe starvation by inducing the expression of those genes involved in the *Strategy II* adaptive mechanism. Microarray results showed the up-regulation of several transcripts involved in methionine cycle (SAMS, MTN, MTK, IDI2, FDH) and in the synthesis and release of MAs (NAS, DMAS, TOM1). The synchronous modulation by Fe deficiency of this pathway was reported in previous works [8, 39]. This response might be correlated with the induction of different IRO transcription factors observed in maize roots (Table 1). The modulation of genes coding for several IRO transcription factors and two formate-dehydrogenase (FDH) isoforms was previously reported in maize roots under low Fe availability [39]; these results strengthen the hypothesis of a strong correlation between Fe deficiency response and the modulation of the enzymes involved in the methionine cycle.

As precursors, SAM metabolites also sustain the synthesis of the phytohormone ethylene, which is induced in *Strategy I* plants and rice by Fe deficiency [45]. Among differentially modulated transcript, data indicated the up-regulation of transcripts coding for ACC synthetase and ACC oxidase, which are directly involved in the synthesis of ethylene. In balance with auxin and cytokinin levels, ethylene might be associated with morphological changes in roots controlling root hair proliferation under Fe deficiency [46]. However the role of ethylene in *Strategy II* plants is still unclear [42, 45]. No evidence of higher ethylene production has been provided in several *Strategy II* plants [45], with the exception of rice, which is characterized by a combined strategy [47]. A clear up-regulation of genes involved in ethylene synthesis has been reported in rice [9], while in maize genes related to the methionine cycle and involved in MAs synthesis have been shown to be induced under Fe-deficiency [42]. Further research on the involvement of ethylene, and its relationships with other hormones, in the morphological and physiological changes occurring in *Strategy II* plants is needed.

Microarray data identified other pathways positively modulated by Fe deficiency in maize roots, such as glycolysis, TCA cycle and mitochondrial chain reactions. Similar transcriptional changes in primary metabolism have been described in many species under Fe deficiency [20, 41, 48]. In particular transcripts coding for an alternative dehydrogenase and an alternative oxidase (AOX1) were strongly induced by Fe starvation in maize roots (present work). As reported by Vigani and Zocchi [49, 50], the induction of these enzymes might be a valid strategy to synthetize ATP bypassing the impairment of the ubiquinone (UQ) reduction process occurring under Fe deficiency.

### Use of different Fe sources by Fe-deficient maize plants

Starting from transcriptional evidence on Fe-deficient roots, we focused our study on the ability of maize plants to use three different natural Fe-sources: Fe-Citrate, Fe-PS or Ferrihydrite, added to the external root solution mimicking condition of low Fe availability [30]. Up to now, the capability of plants to use different Fe-sources occurring in the rhizosphere has been studied mostly in dicots [51].

By real-time RT-PCR the expression of two key genes of *Strategy II* Fe uptake system was monitored up to 24 h of supply: the PS efflux transporter, *ZmTOM1,* and the Fe(III)-PS influx transporter, *ZmYS1*. Both genes were strongly up-regulated in response to Fe deficiency, corroborating microarray data and previous results by other authors [8]. When added to the external solution, Fe was taken up by plants, at levels depending on the solubility of the source. The two transporter genes were strongly down-regulated after 24 h of treatment, independently from the amount of Fe accumulated within the plant; this behaviour was due neither to a circadian rhythm (see expression in -Fe plants, Fig. 5) nor to an over accumulation in the root tissue, but rather involved the overall distribution of Fe within the plants (see uptake and translocation of ^59^Fe, Table 2).

In a recent study Nozoye et al. [8] suggested the involvement of *ZmNRAMP1* (GRMZM2G178190) in the acquisition of external ferrous ions, as already reported for the rice homologous *OsNRAMP1* and *OsNRAMP5* [12–14]. Consistent with this assumption, we found the induction of *ZmNRAMP1* in Fe-deficient maize roots; gene expression was down-regulated by the treatment with the three Fe-sources with a pattern reflecting the amount of ^59^Fe taken up by deficient plants.

Recently, a member of OPT transporter family, AtOPT3 in Arabidopsis [52, 53] and its homologous OsOPT7 in rice [54], has been identified as a responsive element to Fe deficiency. In particular AtOPT3 is a phloem-specific Fe transporter which seems to be essential for the systemic Fe signaling and Fe redistribution within the plant [53]. Transcriptional data of the present work revealed that also in maize roots the homologous gene *ZmOPT7* was induced by Fe deficiency and was strongly down-regulated by the supply of soluble Fe-sources already after 4 h of treatment. These data show that *ZmOPT7*, which is expressed in roots as *OsOPT7*, is highly responsive to Fe availability and possibly involved in Fe homeostasis in maize plants.

After 1 h of treatment Fe-deficient plants acquired more efficiently ^59^Fe from Fe-PS than from the other Fe-sources. This behaviour fits with the mechanism of Fe acquisition in *Strategy II* plants where the Fe-PS complex is the substrate for the YS1 transporter [55]. On the other hand, when Ferrihydrite or Fe-Citrate are provided, Fe needs to be chelated prior to uptake [55, 56]. Nevertheless, after 24 h of ^59^Fe-PS treatment, there was a clear saturation of the amount of Fe accumulated by the root apparatus. This phenomenon could be related to the maintenance of Fe homeostasis, preventing a toxicity effect during Fe-supply to deficient plants [57].

Ferritin gene is involved in Fe storage and in the detoxification of Fe excess [58]. This gene could be considered as an indicator of the iron nutritional status [59], indeed *ZmFerritin* was negatively regulated in Fe-deficient maize roots. Data also provide further evidence that Fe-PS is used efficiently by Fe-deficient plants since already after 4 h of Fe-PS supply, the expression levels of *ZmFerritin* were comparable to those recorded in Fe-sufficient plants.

### Interaction between Fe and P nutrition

Previous studies reported an antagonistic interaction between Fe and P nutrition in rice plants [20]. This behaviour was confirmed in the present work since Fe-deficient maize plants accumulated higher amounts of P as compared to sufficient ones (Fig. 2). This results fits with the up-regulation of the phosphate transporter *ZmPHT1;7* observed in maize roots and its rapid down-regulation upon Fe-supply. The rice homologous of this latter gene (*OsPHT1;6*) plays a general role in the acquisition of inorganic P mediating its high affinity uptake from the rhizosphere and translocation within the plants [60].

It has been shown that low Fe concentration in plant tissues increased the energy demand as well the capacity of oxidative phosphorylation [48]. Moreover P is also an important element for the regulation of a wide range of proteins through post-translational modifications. Recent studies suggest that Fe deficiency induces some changes in the phosphoproteome profile of Arabidopsis roots and this modulation might be linked to a regulative mechanism for Fe homeostasis in plants [61].

Trying to relate Fe uptake with P uptake, ^32^P accumulation was measured in maize roots during supply of different Fe-sources to Fe-deficient plants. Using a soluble P-source (KH_2_^32^PO_4_), ^32^P uptake was lower in Fe-deficient plants than in sufficient ones, particularly when Ferrihydrite was used (Table 3). Furthermore it appeared that levels of ^32^P absorption essentially followed the rates of ^59^Fe uptake in Fe-deficient plants, as it was higher when soluble Fe-sources were supplied. These results suggest that P accumulation might be regulated by Fe uptake in maize plants. In Fe-deficient plants, the low Fe internal concentration might de-repress P uptake leading to an overexpression of the phosphate transporters and a subsequent overaccumulation of P within the tissues. When Fe is supplied a balanced P uptake would occur, possibly avoiding insolubilization of absorbed Fe within the plant tissues. Supporting this idea we also found an overexpression of two transcripts coding for the phosphate transporter PHO1 in Fe-deficient roots. Up-regulation of genes coding for this transporter has been shown to be related to Fe-deficiency response in rice plants [62].

## Conclusions

Results of the present work show that besides the well-characterized response to Fe deficiency at physiological and transcriptional levels, maize roots also show morphological modifications of root system and up-regulation of transcripts usually found in *Strategy I* plants [63]. Particularly a role of ethylene and other hormones and ferrous ion transporter would deserve further research.

Furthermore, further evidence for a close relationship between Fe and P homeostasis have been provided that could help shedding light on the reciprocal regulation in the uptake and distribution of the two essential elements in maize plants.

## Additional files

Additional file 1: Figure S1. SPAD index values of leaf tissues were measured on 12, 15 and 19-day-old maize plants grown under Fe-deficient- or Fe-sufficient-condition (−Fe and + Fe plants, respectively). **Figure S2.** External acidification of maize roots under Fe deficiency (B, D) and Fe sufficiency (A, C). **Figure S3.** Functional distribution among Gene Ontology (GO) categories of up- and down-regulated transcripts differentially modulated by Fe deficiency (−Fe *vs* + Fe transcriptomic comparison). **Figure S4.** Overview of up- (A) and down- (B) modulated transcripts in -Fe vs + Fe comparison using MapMan-bincode classification. **Figure S5.** Iron-(^59^Fe) accumulated in maize leaves (A) and roots (B). **Figure S6.** Phosphorous-(^32^P) accumulated in maize leaves (A) and roots (B). (PDF 6393 kb)
Additional file 2: Table S1. Morphometric evaluation of maize roots in response to Fe deficiency. (PDF 51 kb)
Additional file 3: Table S2. Differentially expressed transcripts resulted by the comparison of root transcriptional profiles of Fe-deficient plants with root transcriptional profile of Fe-sufficient ones (−Fe *vs* + Fe). (XLSX 160 kb)

## Abbreviations

EDTA: Ethylenediaminetetraacetic acid
epi-HMA: Epi-hydroxymugineic acid
MES: 2-(N-morpholino) ethanesulfonic acid
