# Supplementary material for: Transcriptional and physiological analyses of Fe deficiency response in maize reveal the presence of Strategy I components and Fe/P interactions
Source: BMC Genomics. 2017 Feb 13;18:154. doi: 10.1186/s12864-016-3478-4 (PMC5307951; doi:10.1186/s12864-016-3478-4)
Supplement: Additional file 1: Figure S1. — SPAD index values of leaf tissues were measured on 12, 15 and 19-day-old maize plants grown under Fe-deficient- or Fe-sufficient-condition (−Fe and + Fe plants, respectively). Figure S2. External acidification of maize roots under Fe deficiency (B, D) and Fe sufficiency (A, C). Figure S3. Functional distribution among Gene Ontology (GO) categories of up- and down-regulated transcripts differentially modulated by Fe deficiency (−Fe vs + Fe transcriptomic comparison). Figure S4. Overview of up- (A) and down- (B) modulated transcripts in -Fe vs + Fe comparison using MapMan-bincode classification. Figure S5. Iron-(59Fe) accumulated in maize leaves (A) and roots (B). Figure S6. Phosphorous-(32P) accumulated in maize leaves (A) and roots (B). (PDF 6393 kb) [file 12864_2016_3478_MOESM1_ESM.pdf]

ADDITIONAL FILE 1

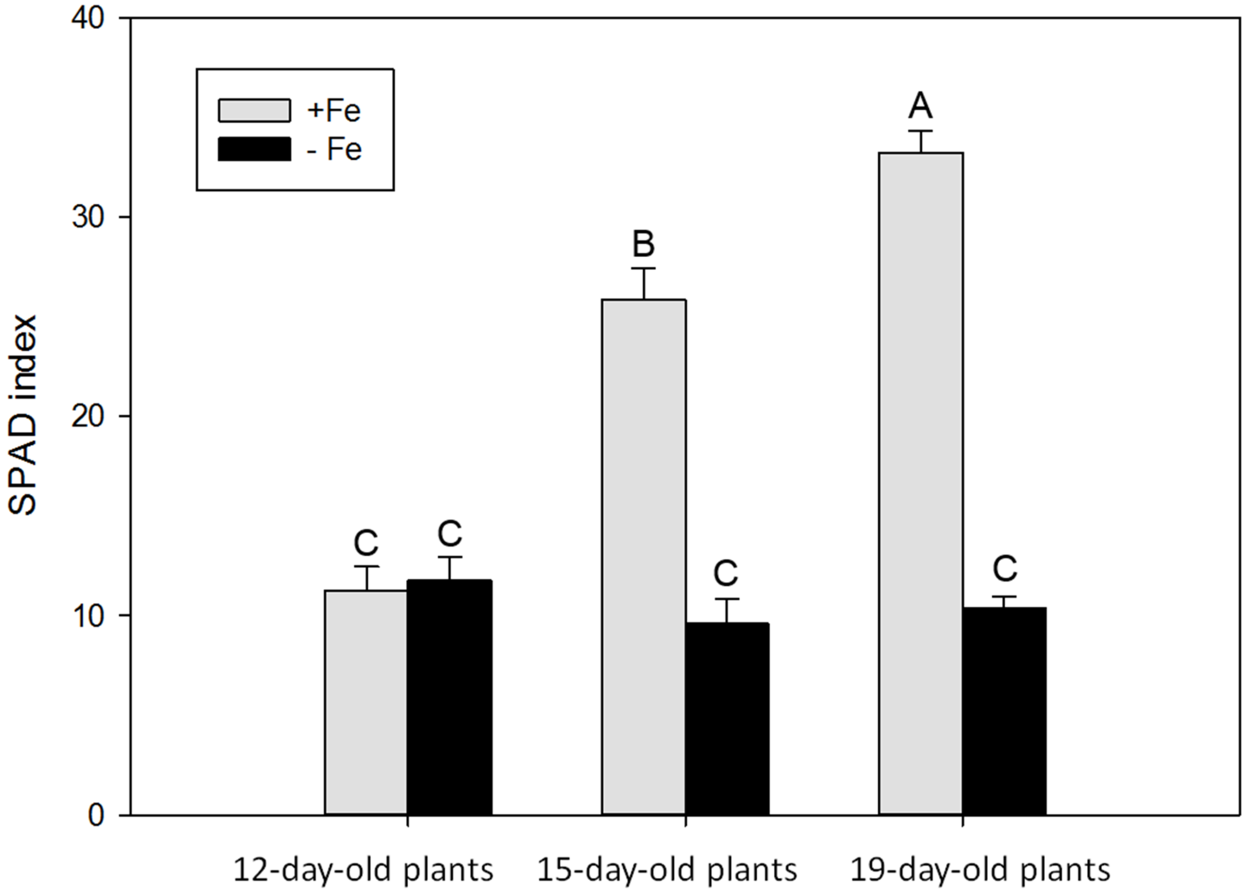

Figure S1. SPAD index values of leaf tissues were measured on 12, 15 and 19-day-old maize plants grown under Fe-deficient- or Fe-sufficient-condition (-Fe and +Fe plants, respectively). Data are means ± SD of three independent experiments (capital letters refer to statistically significant differences among the mean, ANOVA Holm–Sidak, $P < 0.05$, $n = 3$).

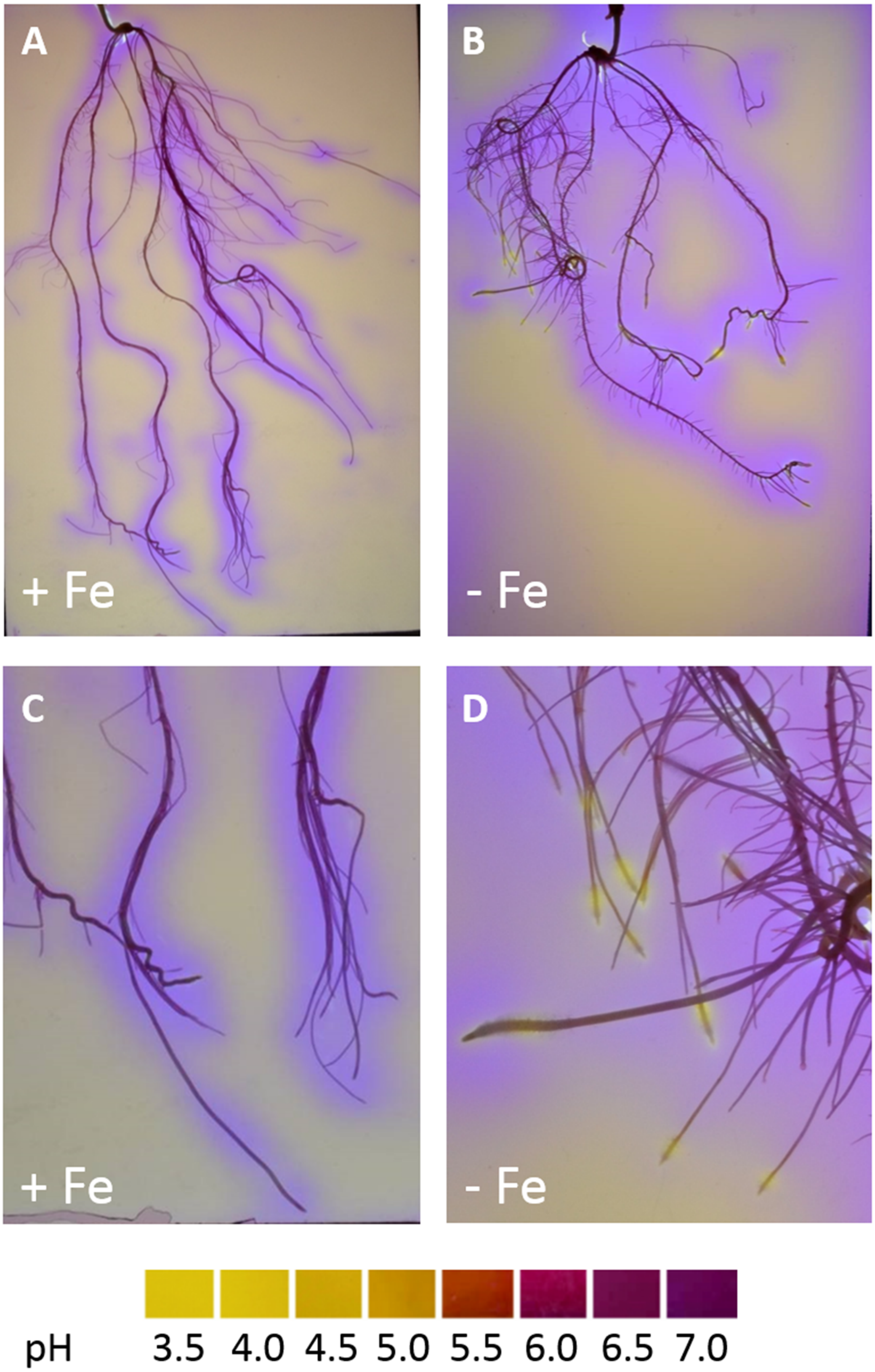

Figure S2. External acidification of maize roots under Fe deficiency (**B, D**) and Fe sufficiency (**A, C**). Maize roots were imbedded for 4 hours in agar gel containing pH indicator (Bromocresol purple); yellow indicates acidification of agar gel (pH< 5.5) and purple indicates an alkalization above pH 7.

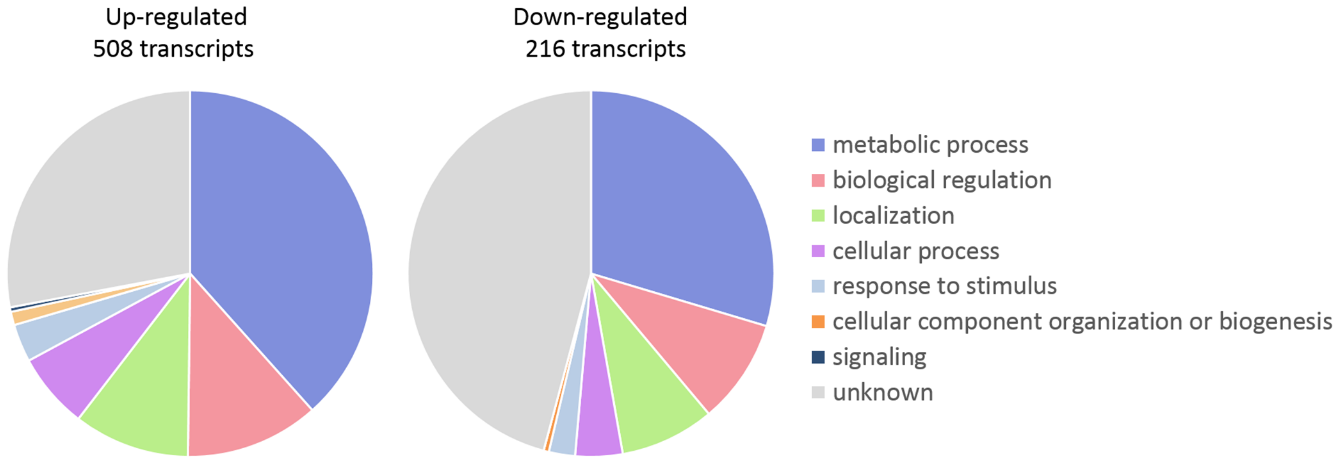

Figure S3. Functional distribution among Gene Ontology (GO) categories of up- and down-regulated transcripts differentially modulated by Fe deficiency (-Fe *vs* +Fe transcriptomic comparison). Transcripts were clustered according to the terms of "biological process" categories of GO (FC $\geq |1.5|$, adjusted P-value ≤ 0.05, n = 3).

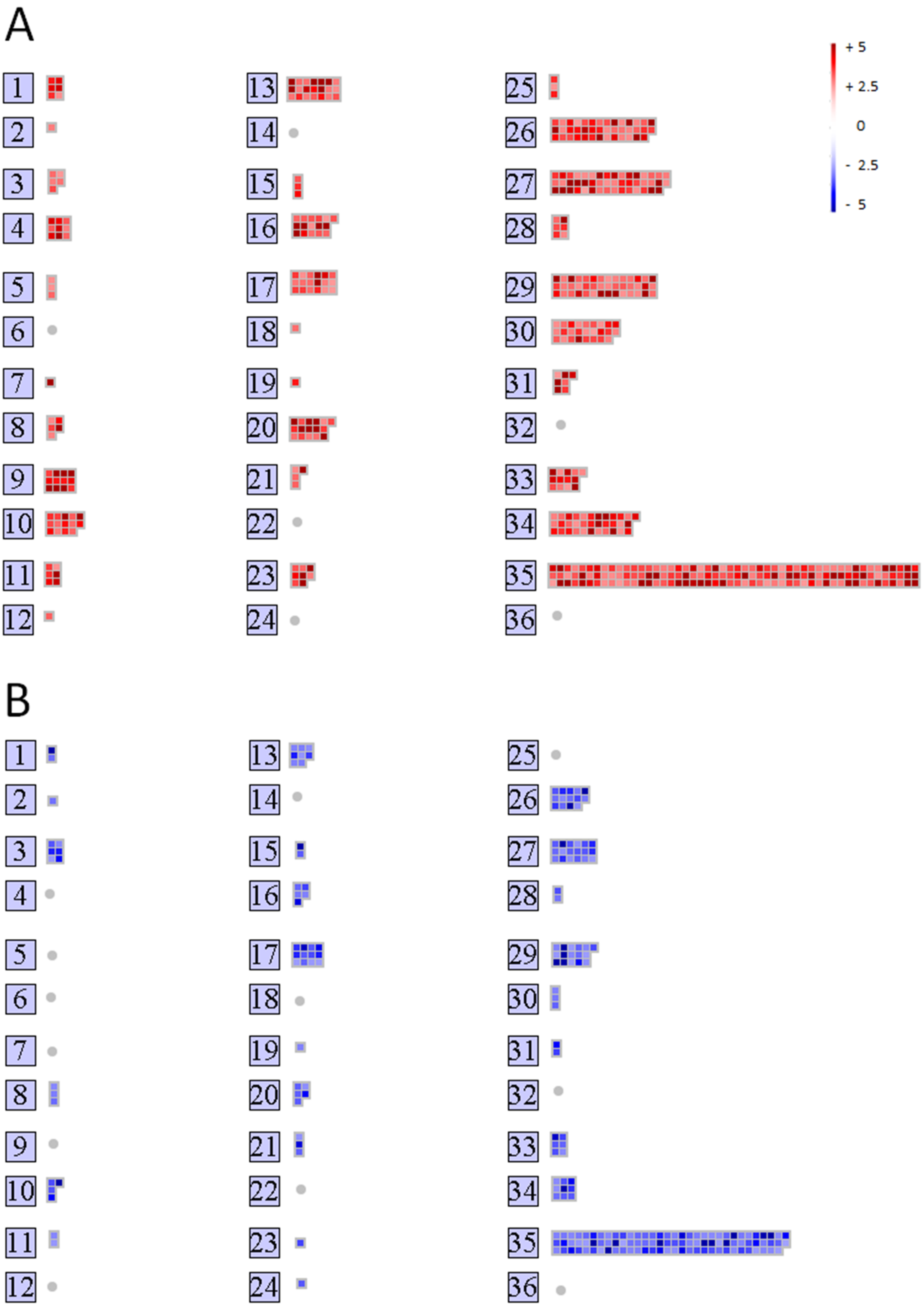

Figure S4. Overview of up- (**A**) and down- (**B**) modulated transcripts in -Fe vs +Fe comparison using MapMan-bincode classification. Numbers refer to the MapMan bincode: 1, PS; 2, major CHO

metabolism; 3, minor CHO metabolism; 4, glycolysis; 5, fermentation; 6, gluconeogenesis / glyoxylate cycle; 7, OPP; 8, TCA / org transformation; 9, mitochondrial electron transport / ATP synthesis; 10, cell wall; 11, lipid metabolism; 12, N-metabolism; 13, amino acid metabolism; 14, S-assimilation; 15, metal handling; 16, secondary metabolism; 17, hormone metabolism; 18, Co-factor and vitamine metabolism; 19, tetrapyrrole synthesis; 20, stress; 21, redox; 22, polyamine metabolism; 23, nucleotide metabolism; 24, Biodegradation of Xenobiotics; 25, C1-metabolism; 26, miscellaneous; 27, RNA; 28, DNA; 29, protein; 30, signalling; 31, cell; 32,micro RNA, natural antisense; 33, development; 34, transport; 35, not assigned. Color scale refers to the fold change values of differentially expressed transcripts: red color refers to those transcripts positively regulated by Fe deficiency, while in blue are transcripts negatively regulated by Fe deficiency ($FC \geq |1.5|$, adjusted P-value ≤ 0.05, $n = 3$).

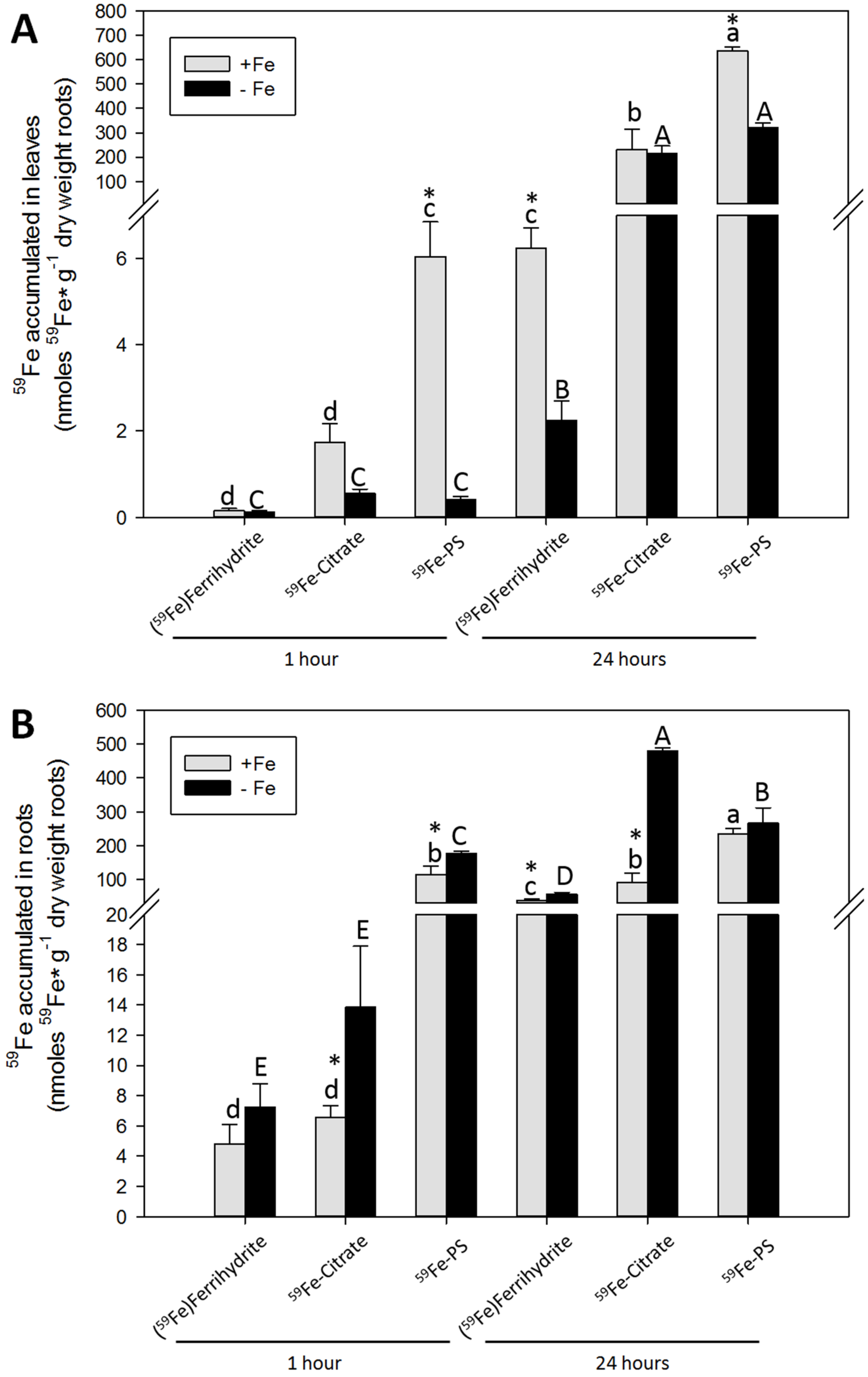

Figure S5. Iron-(^{59}Fe) accumulated in maize leaves (**A**) and roots (**B**). The plant ability to accumulate Fe was evaluated by ^{59}Fe uptake experiments on Fe-sufficient (+Fe, grey bars) and Fe-deficient (-Fe, black bars) plants treated for 1 and 24 hours with three labelled ^{59}Fe-sources: (^{59}Fe)Ferrihydrite, ^{59}Fe-

Citrate or ^{59}Fe-PS. Iron-(^{59}Fe) was added to nutrient solution at final concentration of 1 μM. Data are means + SD of three independent experiments. *Small letters,* refer to statistically significant differences among Fe-sufficient plants; *capital letters,* refer to statistically significant differences among Fe-deficient plants, *asterisks*, refer to statistically significant differences between the two growth condition (-Fe and +Fe). ANOVA Holm–Sidak, $P < 0.05$, $n = 3$). DW, dry weight.

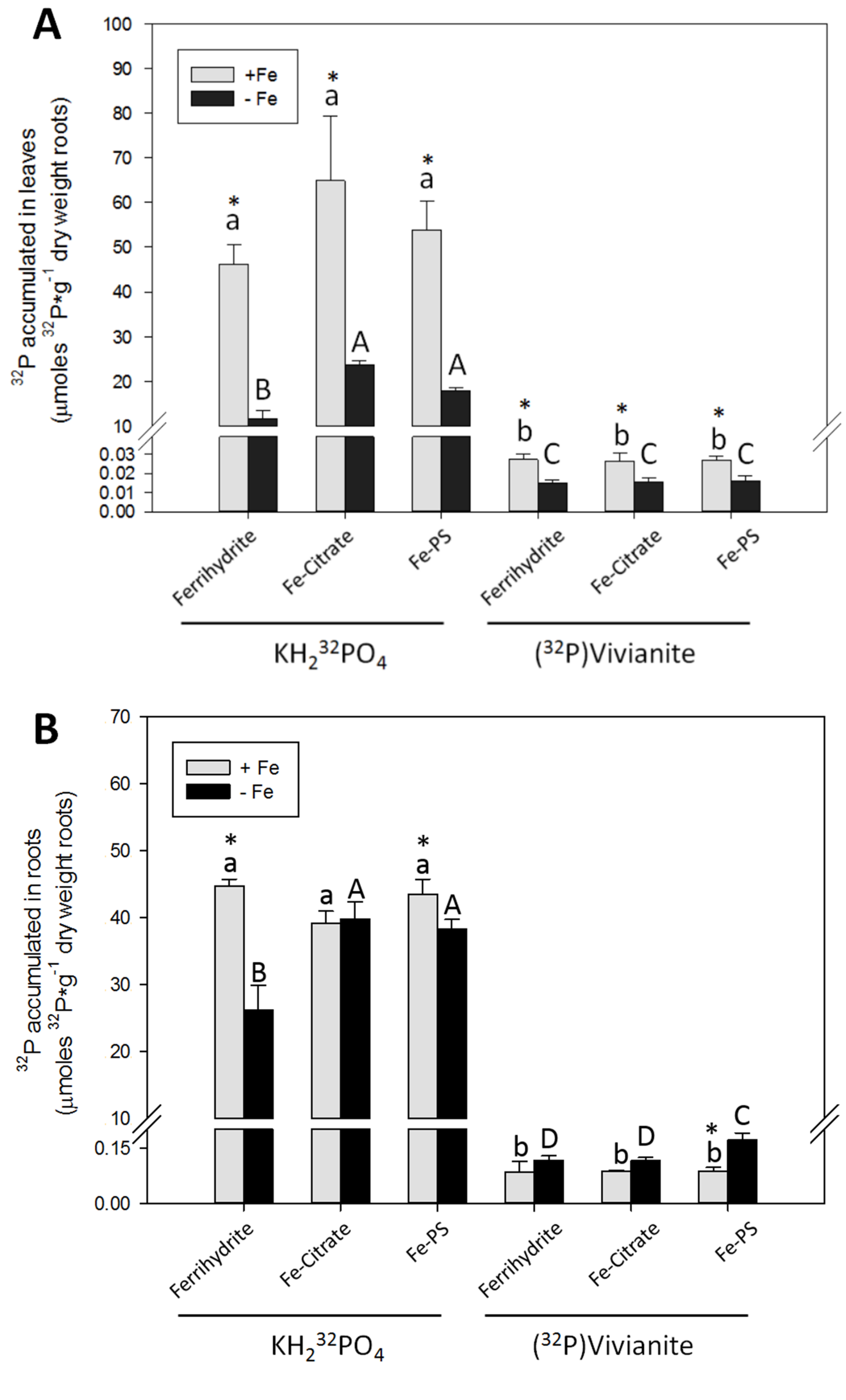

Figure S6. Phosphorous-(^{32}P) accumulated in maize leaves (**A**) and roots (**B**). The plant ability to accumulate P was evaluated by ^{32}P uptake experiments on Fe-sufficient (+Fe, grey bars) and Fe-

deficient (-Fe, black bars) plants. Up to 24 hours, -Fe and +Fe plants were treated with three unlabelled Fe-sources (Ferrihydrite, Fe-Citrate or Fe-PS; 1 µM Fe) provided in conjunction with two different labelled ^{32}P-sources : $KH_2{}^{32}PO_4$ or (^{32}P)Vivianite (175 µM P). Data are means+SD of three independent experiments. *Small letters,* refer to statistically significant differences among Fe-sufficient plants; *capital letters,* refer to statistically significant differences among Fe-deficient plants, *asterisks*, refer to statistically significant differences between the two growth condition (-Fe and +Fe). ANOVA Holm–Sidak, $P < 0.05$, $n = 3$). DW, dry weight.
