# Supplementary material for: Transcriptional and physiological analyses of Fe deficiency response in maize reveal the presence of Strategy I components and Fe/P interactions
Source: BMC Genomics. 2017 Feb 13;18:154. doi: 10.1186/s12864-016-3478-4 (PMC5307951; doi:10.1186/s12864-016-3478-4)
Supplement: Additional file 2: Table S1. — Morphometric evaluation of maize roots in response to Fe deficiency. (PDF 51 kb) [file 12864_2016_3478_MOESM2_ESM.pdf]

ADDITIONAL FILE 2

Table S1: Morphometric evaluation of maize roots in response to Fe deficiency.
Representative pictures of Fe-deficient (-Fe) and Fe-sufficient (+Fe) root systems are shown in **Figure 1**. The analyses of root systems were performed using "GiA Roots" software [63]. Trait descriptions are following [63]: *Maximum Number of Roots*, after sorting the number of roots crossing a horizontal line from smallest to largest, the maximum number is considered to be the 84th-percentile value (one standard deviation); *Network Perimeter* (cm), the total number of network pixels connected to a background pixel; *Network Area* (cm^2), the number of network pixels in the image; *Network Surface Area* (cm^2), the sum of the local surface area at each pixel of the network skeleton, as approximated by a tubular shape whose radius is estimated from the image; *Network Length* (cm), the total number of pixels in the network skeleton; *Network Volume* (cm^3), the sum of the local volume at each pixel of the network skeleton, as approximated by a tubular shape whose radius is estimated from the image; the "GiA Roots" software converted pixel data in cm unit. *Primary Root Length* (cm), obtained by *in vivo* measurements. Data are means ± SD based on three independent biological replicates (*asterisks* refer to statistically differences between –Fe and +Fe plants, Student-Newman-Keuls Method ANOVA, $n = 3$, $P < 0.05$).

	Maximum Number of Roots	Network Perimeter<br>cm	Network Area<br>cm^2	Network Surface Area<br>cm^2	Network Length<br>cm	Network Volume<br>cm^3	Primary Root Length<br>cm
**+ Fe**	79.3 ± 1.5	3,054.5 ± 205.8	40.0 ± 2.8	141.8 ± 9.9	1,448.5 ± 92.2	1.25 ± 0.09	22.72 ± 1.86
**- Fe**	72.5 ± 3.0 *	2,508.5 ± 121.8 *	32.6 ± 1.8 *	114.6 ± 6.8 *	1,168.3 ± 54.2 *	1.01 ± 0.09 *	14.63 ± 1.37 *
